# Properties of SiCN Films Relevant to Dental Implant Applications

**DOI:** 10.3390/ma16155318

**Published:** 2023-07-28

**Authors:** Xinyi Xia, Chao-Ching Chiang, Sarathy K. Gopalakrishnan, Aniruddha V. Kulkarni, Fan Ren, Kirk J. Ziegler, Josephine F. Esquivel-Upshaw

**Affiliations:** 1 Department of Chemical Engineering, College of Engineering, University of Florida, Gainesville, FL 32611, USA; xiaxinyi@ufl.edu (X.X.);; 2 Department of Restorative Dental Sciences, Division of Prosthodontics, College of Dentistry, University of Florida, Gainesville, FL 32610, USA

**Keywords:** surface coating, dental implants, ternary silicon carbon nitride (SiCN), PECVD film deposition, chemical composition, optical, tribological properties

## Abstract

The application of surface coatings is a popular technique to improve the performance of materials used for medical and dental implants. Ternary silicon carbon nitride (SiCN), obtained by introducing nitrogen into SiC, has attracted significant interest due to its potential advantages. This study investigated the properties of SiCN films deposited via PECVD for dental implant coatings. Chemical composition, optical, and tribological properties were analyzed by adjusting the gas flow rates of NH_3_, CH_4_, and SiH_4_. The results indicated that an increase in the NH_3_ flow rate led to higher deposition rates, scaling from 5.7 nm/min at an NH_3_ flow rate of 2 sccm to 7 nm/min at an NH_3_ flow rate of 8 sccm. Concurrently, the formation of N-Si bonds was observed. The films with a higher nitrogen content exhibited lower refractive indices, diminishing from 2.5 to 2.3 as the NH_3_ flow rate increased from 2 sccm to 8 sccm. The contact angle of SiCN films had minimal differences, while the corrosion rate was dependent on the pH of the environment. These findings contribute to a better understanding of the properties and potential applications of SiCN films for use in dental implants.

## 1. Introduction

The application of surface coatings is a widely used technique that significantly enhances the performance and prolongs the lifespan of medical and dental implants, making them indispensable in the field of implantology [1,2]. Despite titanium’s long-standing success as a dental implant material, recent developments have brought potential replacements to light [3,4,5]. Consequently, the development of surface coatings that can enhance the performance and durability of dental implants has become a significant area of research [6,7,8].

One of the main challenges associated with dental implants is the risk of infection. Researchers have been working on developing antibacterial coatings for dental implants to address this issue [4,9,10]. These coatings can inhibit the growth of bacteria on the surface of the implant, reducing the risk of infection and improving implant success rates [11]. Hydrophilic coatings can attract water molecules, which improves the implant’s ability to integrate with the surrounding bone tissue. Bioactive coatings are designed to stimulate the growth of new bone tissue around the implant, improving its stability and reducing the risk of implant failure [12]. Nanocoatings are another area of development in dental implant coatings. These coatings are made up of extremely small particles that can be designed to have specific properties, such as antibacterial or bioactive properties [13].

Silicon carbide (SiC) as a dental implant coating material has gained attention due to its exceptional properties, such as its high hardness and wear resistance, leading to a durable and long-lasting coating [14,15]. In addition, SiC demonstrates biocompatibility, exhibiting no deleterious effects when in contact with living tissue. Moreover, SiC coatings exhibit antibacterial properties, which have the potential to reduce the risk of infection in the area surrounding the implant [12].

Ternary silicon carbon nitride (SiCN), obtained by introducing nitrogen into silicon carbide (SiC), has attracted significant interest due to its potential advantages [16,17]. The properties of SiCN coatings can be customized by adjusting the ratio of silicon, carbon, and nitrogen in the coating. This allows the coating to be tailored to specific dental implant applications, providing improved properties and performance. Nitrogen-containing compounds exhibit antibacterial properties [18,19]. Although the antibacterial mechanism of SiCN coatings is not yet fully understood, several potential mechanisms have been proposed. The introduction of nitrogen into the SiC coating to form SiCN may decrease the surface’s attractiveness to bacteria, which may lead to a reduction in bacterial colonization and infection risk. Additionally, nitrogen-containing compounds may have an inhibitory effect on bacterial growth [20,21].

Studies have investigated the potential of SiCN as a coating material for joint replacement implants [18,22,23]. Various deposition techniques, including ion beam sputtering assisted deposition [18], high power impulse magnetron sputtering (HiPIMS) [23], thermal chemical vapor deposition (CVD) [22], and plasma-enhanced CVD (PECVD) have been employed to synthesize SiCN coatings [24,25]. Among these processes, PECVD is the preferred technique due to its ability to provide highly conformal coatings with excellent uniformity over complex geometries [26]. In addition, PECVD offers low-temperature processing, thereby minimizing thermal damage to the implant or temperature-sensitive components [27]. Precise control over the deposition parameters enables tailoring of coating properties, such as thickness, composition, and porosity, to meet specific application requirements [28]. However, few studies have utilized PECVD to develop SiCN coatings for medical or dental implant applications.

This study aims to investigate the chemical composition, optical properties, and tribological properties of SiCN films deposited via PECVD by adjusting the gas flow rates of ammonia (NH_3_), methane (CH_4_), and silane (SiH_4_) to vary the content concentrations. The SiCN films’ surface composition, atomic concentration, and chemical bonds were analyzed to obtain insights into the coating’s properties. The refractive index was measured to determine the optical properties, while the wettability contact angle was assessed to evaluate the potential antibacterial ability. Additionally, the corrosion rates in different pH solutions (pH 2, pH 7, and pH 10) were studied to assess the coating’s suitability for dental applications.

## 2. Materials and Methods

### 2.1. Sample Preparation

The same preparation procedures were used for both the silicon substrate before applying the coatings and the finished samples prior to characterization. The samples were thoroughly cleaned with acetone and isopropyl alcohol, followed by blow-drying with nitrogen. Next, the surfaces were treated with ozone to remove any traces of adventitious carbon.

### 2.2. SiCN Coating

In this study, a plasma-enhanced chemical vapor deposition system (PECVD SLR Series, Plasma-Therm, Saint Petersburg, FL, USA) was used to deposit SiCN onto silicon substrates. The system consisted of a parallel plate configuration, gas shower head, and load lock. NH_3_, CH_4_, and SiH_4_ were used as gas precursors for SiCN deposition. The deposition temperature was set to 350 °C, and three sets of experiments were performed with varying gas flow rates for each gas species. The radio frequency (RF) power was set to 50 W and operated at a frequency of 13.56 MHz. The chamber pressure was set to 900 mTorr.

To verify the deposition rate, SiCN was deposited onto reference wafers, and the total thickness was measured using a surface profilometer (Alpha-Step 500, KLA-Tencor, Milpitas, CA, USA).

### 2.3. Characterization Techniques

#### 2.3.1. X-ray Photoelectron Spectroscopy (XPS) Surface Composition Analysis

The surface composition of the SiCN for all conditions studied was determined using a Physical Instruments ULVAC PHI XPS system (ULVAC-PHI, Kanagawa, Japan) and CasaXPS. A source power of 300 W from a monochromatic Al X-ray source (1486.6 eV) was used, along with an electron pass energy of 93.5 eV for the survey scans. The acceptance angle was set at 7°, the take-off angle at 50°, and the analysis spot diameter at 100 µm. The binding energy accuracy was within 0.03 eV, while the overall energy resolution of the XPS was approximately 0.1 eV. Charge correction was carried out using the C-Si peak at 283.5 eV.

#### 2.3.2. Deposition Rate Determination

The deposition rates were measured using a surface profilometer (Alpha-Step 500, KLA-Tencor, Milpitas, CA, USA) and calculated. To ensure accurate measurement, part of the substrate area was covered with a glass slide during film deposition. After the deposition and removal of the glass slide, the height difference at the edge of the film was measured using the surface profilometer, and the deposition rate was calculated by dividing the deposition time.

#### 2.3.3. Refractive Index Measurement

The refractive index, a critical parameter in assessing the optical properties of the SiCN films, was determined using a Filmetrics F40 photospectrometer (F40, Filmetrics, San Diego, CA, USA). This instrument, which is known for its high-accuracy measurements of thin-film optical properties, operates by directing light onto the film and quantifying the light reflected back. The resulting data allows for the calculation of the refractive index, indicating how significantly light slows down (or refracts) in the SiCN film compared to a vacuum. This measure provides essential insights into the suitability and potential performance of the SiCN films when used in specific applications such as medical and dental implants.

#### 2.3.4. Contact Angles Measurement

Contact angle measurements serve as a key indicator of wettability, which has implications for the adhesion and tribological properties of the SiCN films. These measurements were carried out using a Kruss DSA100 Drop Shape Analyzer (Hamburg, Germany). This instrument, well regarded for its precision, applies a droplet of liquid to the film surface and then utilizes high-resolution imaging to analyze the shape of the droplet. The angle formed at the liquid-film interface—the contact angle—is then precisely determined. This measure provides valuable information about the surface characteristics of the SiCN films.

#### 2.3.5. Corrosion Rate Determination

Assessing the corrosion rate of SiCN films under different pH conditions is crucial to understanding their durability and performance in a range of environments. In this experiment, samples were exposed to three distinct buffer solutions with pH levels of 2, 7, and 10, simulating varying acidic, neutral, and basic conditions. Each sample was immersed in its respective solution for a duration of eight hours, after which the extent of corrosion was measured using a surface profilometer. This instrument, used for its ability to accurately gauge surface profile changes, helped determine the corrosion rate of the SiCN films. This information is essential to predict the longevity and stability of the SiCN films, particularly when applied to medical and dental implants which may be exposed to different pH environments.

## 3. Results and Discussion

**Figure 1:**
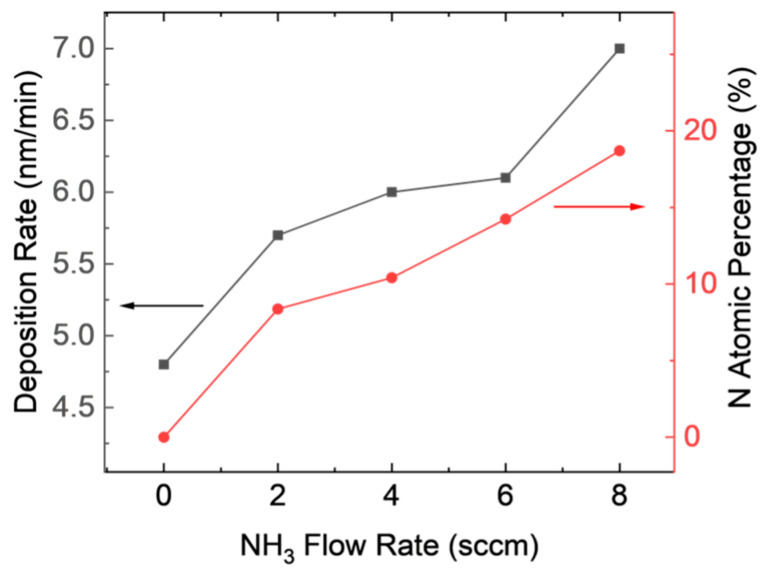
Deposition rate and N atom percentage for SiCN film with varying NH_3_ flow rates.

Figure 1 shows that the deposition rate slightly increased with increasing NH_3_ flow rate in the experiments with varying NH_3_ flow rates. The flow rates of SiH_4_ and CH_4_ were selected to match those used in previous studies on SiC film formation. The resulting deposition rate of the SiCN film is on the scale of nanometers per minute, which enables fine control over the film thickness for thin film applications. In comparison, the established methods for SiC and SiN coatings for dental implants are typically in the range of 200–500 nm [12,15,29,30]. By depositing a SiCN film with a similar thickness using this process, it is feasible to achieve a high-quality film in a reasonable time frame.

**Figure 2:**
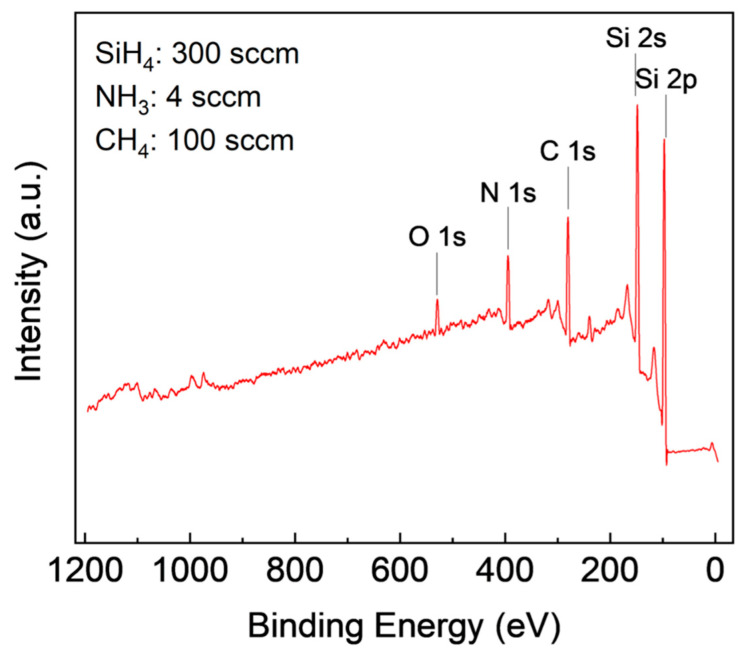
XPS survey scans of SiCN film deposited with 300 sccm SiH_4_, 4 sccm NH_3_, and 100 sccm CH4.

XPS is a surface analysis technique that provides elemental and chemical composition information of a material. Analysis of the XPS spectrum involves identifying the characteristic peaks of the elements present in the sample to determine its chemical composition. The peak position on the XPS spectrum provides information on the chemical and oxidation state of the elements [31]. In this study, CasaXPS software was used to analyze the film’s composition, where Gaussian curve fitting was applied to identify peaks, and the area under the curve was used to calculate atomic concentration. The survey scan in Figure 2 showed the presence of Si, C, and N in the film, and the presence of oxygen is due to surface oxidation.

XPS can also be used to identify chemical bonds present in a material by analyzing the binding energy of the electrons that are emitted from the sample after X-ray irradiation [31]. Spectral deconvolution is a common method used to identify chemical bonds in XPS analysis, which separates the contributions of each chemical bond to the overall spectrum. In this study, OriginLab software was utilized to plot and deconvolute the XPS data. The deconvolution technique was performed to identify the chemical shifts from the XPS spectra, and the C-Si peak at 283.5 eV was utilized for charge referencing [32].

**Figure 3:**
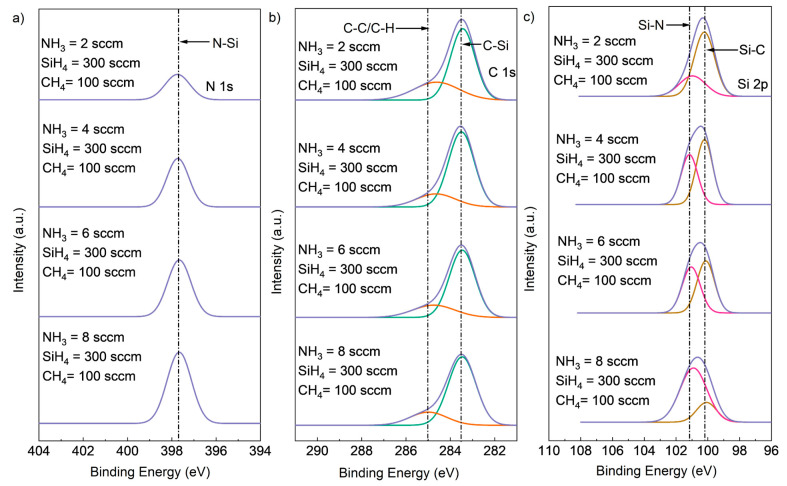
Deconvoluted high-resolution XPS spectra of (**a**) Si2p, (**b**) C1s (deconvoluted into two peaks, C-Si (green peak), C-C or C-H (red peak)), and (**c**) N1s core-level peaks (deconvoluted into two peaks, Si-N (red peak) and Si-C (brown peak)) for SiCN deposited with different NH_3_ flow rates.

The results of XPS composition analysis with different NH_3_ flow rates are shown in Table 1. The N content increased from 7.37% to 17.50% with an increasing NH_3_ flow rate from 2 sccm to 8 sccm, while the Si and C content decreased. The peak located at 398 eV corresponded to the N-Si peak, and its intensity increased in direct correlation with the NH_3_ flow rate. This suggests that the increase in NH_3_ flow rate increased the formation of N-Si bonds while attenuating the formation of Si-C bonds. The high-resolution XPS spectra of Si2p, C1s, and N1s core-level peaks for SiCN deposited with different NH_3_ flow rates are shown in Figure 3. The main peaks were deconvoluted into peaks according to different chemical bonds, with different bonds having different binding energies. The peak located at 397.7 eV for the N1s spectrum was likely to be the N-Si bonds. The C1 peak was further deconvoluted into C-C or C-H bonds located at 285 eV and a C-Si bonds located at 283.5 eV. Two peaks were deconvoluted from Si2p, with the peak at 101.2 corresponding to Si-N bonds and the peak at 100.3 corresponding to Si-C bonds. The N-Si peak intensity for N1s increased with increasing NH_3_ flow rate from 2 sccm to 8 sccm, while the Si-N peak intensity for Si2p increased, and the Si-C peak intensity for Si2p decreased. This is consistent with the atomic ratio result in Table 1, where more N-Si bonds were formed when the NH_3_ flow rate increased from 2 sccm to 8 sccm.

**Figure 4:**
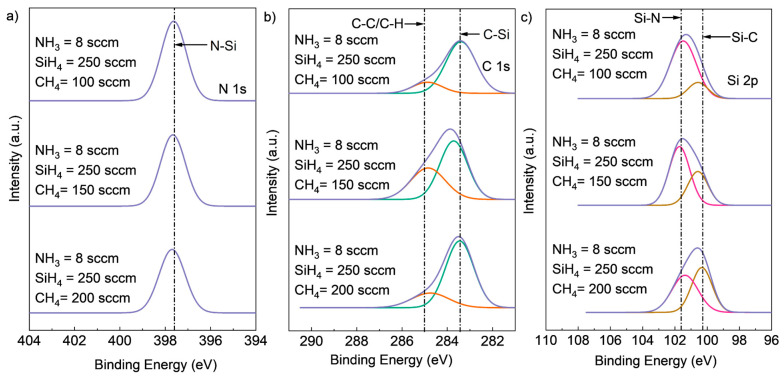
Deconvoluted high-resolution XPS spectra of (**a**) Si2p, (**b**) C1s (deconvoluted into two peaks, C-Si (green peak), C-C or C-H (red peak)), and (**c**) N1s core-level peaks (deconvoluted into two peaks, Si-N (red peak) and Si-C (brown peak)) for SiCN deposited with different CH_4_ flow rates.

To investigate the effect of varying CH_4_ flow rates on the chemical composition of SiCN films, experiments were conducted with fixed NH_3_ flow rates of 8 sccm and fixed SiH_4_ flow rates of 250 sccm, while the CH_4_ flow rate was changed from 100 sccm to 150 sccm and 200 sccm. The resulting high-resolution XPS spectra and atomic concentration of Si2p, C1s, and N1s core-level peaks for the deposited SiCN films are shown in Figure 4 and Table 1. As expected, an increase in CH_4_ flow rate led to a higher carbon content and lower N content in the SiCN films. Specifically, the peak intensity of the C-Si chemical bonding peak in the C1s spectrum and the Si-C bonding peak in the Si2p spectrum increased with an increasing CH_4_ flow rate. Meanwhile, the intensity of the N-Si peak in the N1s spectrum and Si-N peak in the Si2p spectrum decreased. These observations are consistent with the atomic concentration results presented in Table 1.

**Figure 5:**
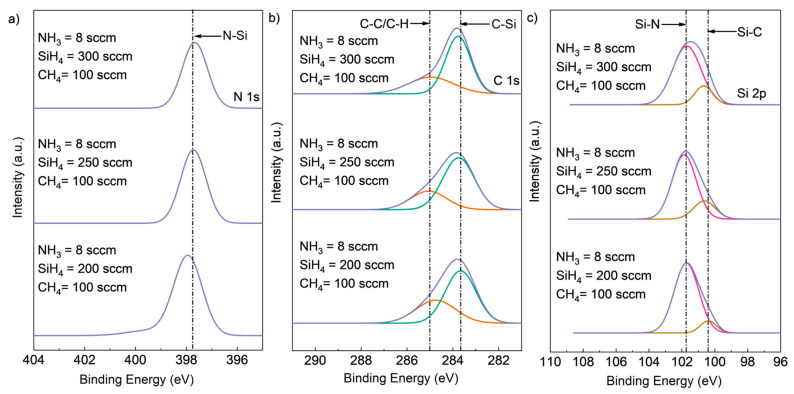
Deconvoluted high-resolution XPS spectra of (**a**) Si2p, (**b**) C1s (deconvoluted into two peaks, C-Si (green peak), C-C or C-H (red peak)), and (**c**) N1s core-level peaks (deconvoluted into two peaks, Si-N (red peak) and Si-C (brown peak)) for SiCN deposited with different SiH_4_ flow rates.

To investigate the effect of varying SiH_4_ flow rates on the chemical composition of SiCN films, experiments were conducted with fixed NH_3_ flow rates of 8 sccm and fixed CH_4_ flow rates of 100 sccm. In Figure 5, as the SiH_4_ flow rate was decreased, the Si composition of the films also decreased, as evidenced by the decreased intensity of the C-Si peak in the C1s spectrum and the Si-C peak in the Si2p spectrum. No significant decrease in the peak intensity of N-Si in N1s or Si-N in Si2p was observed, indicating that Si-N bond formation was unaffected by the change in SiH_4_ flow rate. However, a higher intensity of the C-C/C-H bonds peak was found in the C1s spectrum, suggesting increased formation of these bonds with decreasing SiH_4_ flow rate.

The nitrogen atoms in SiCN films can create a negatively charged surface, which can interact with positively charged sites on bacterial cell membranes, leading to membrane disruption and cellular damage [33,34]. Additionally, SiCN films can generate reactive oxygen species (ROS) upon exposure to light or heat, which can further damage bacterial cells [35,36].

The negative charge on SiCN surfaces is primarily generated through the formation of N-Si bonds, which results from the presence of nitrogen atoms in the material. In the N-Si bond, the nitrogen atom donates a lone pair of electrons to form a covalent bond with the silicon atom, creating a negatively charged nitrogen atom and a positively charged silicon atom. This negatively charged nitrogen atom can interact with positively charged sites on bacterial cell membranes, leading to membrane disruption and cellular damage [37,38,39,40].

In this study, we found that the NH_3_ flow rate plays a significant role in the formation of N-Si bonds in SiCN films. Furthermore, N-Si bonds were present in all of the examined SiCN films. These findings provide insight into the mechanism underlying the antibacterial properties of SiCN films and have implications for their potential use in medical and dental applications.

**Figure 6:**
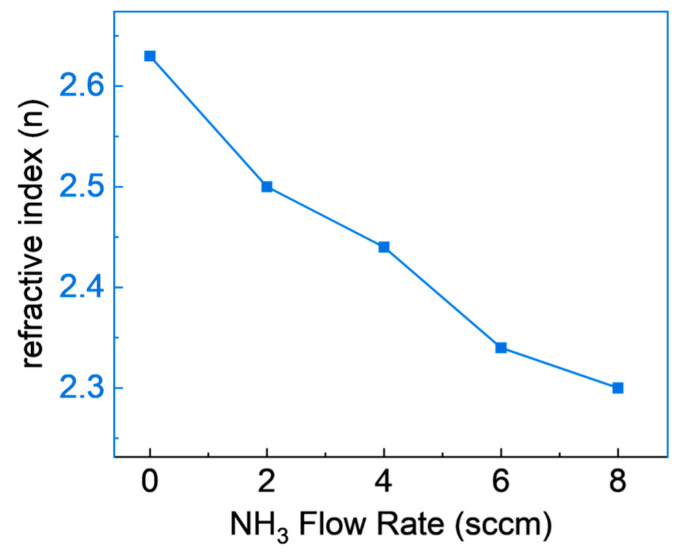
Refractive indices of SiCN films deposited with various NH_3_ flow rates.

Figure 6 depicts the refractive indices of SiCN films produced under varying NH_3_ flow rates, specifically within a range of 2 to 8 cm. These refractive indices were determined utilizing a photospectrometer at a wavelength of 632 nm. An inverse relationship between the NH_3_ flow rate and the refractive index of the SiCN films is noticeable, with an increase in the NH_3_ flow rate leading to a decrease in the refractive index. This phenomenon is likely due to the increased integration of nitrogen (N) into the film with elevated NH_3_ flow rates, as validated by the high-resolution XPS data contained in Table 1 and Figure 3. The observed shift in refractive index carries significant implications for the practical uses of SiCN films. High refractive indices can enhance the optical properties of dental coatings, increasing the brightness and translucency of tooth-colored restorations, a finding supported by Chen et al., 2019 [41]. Conversely, an increase in the refractive index might contribute to an increase in coating brittleness, rendering them more prone to cracking or chipping, as reported by Pan et al., 2014 [42]. Such vulnerability may negatively impact the material’s long-term performance and durability. Consequently, the optimal refractive index for dental coatings may be application-specific and require a careful balance between optical and mechanical properties. Future investigations should aim at determining this optimal balance to unlock the full potential of SiCN films in dental applications.

**Figure 7:**
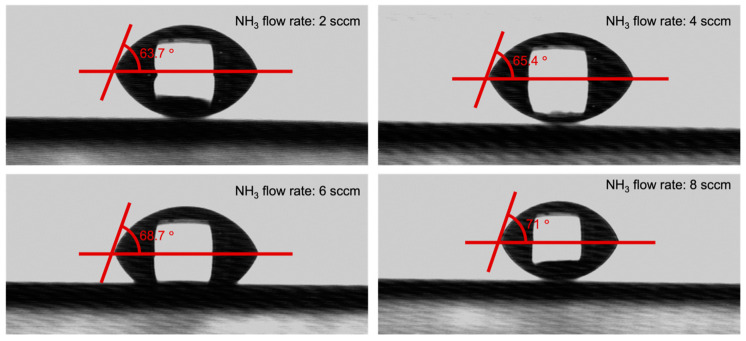
Contact angles of SiCN films deposited with various NH_3_ flow rates.

Figure 7 illustrates the impact of NH_3_ flow rate on the wettability of SiCN. The SiCN film deposited with a higher NH_3_ flow rate displayed slightly larger contact angles, ranging from 63.7° at 2 sccm to 71° at 8 sccm. However, the change in contact angle was relatively small. An excessively hydrophobic surface can repel water and create a barrier that prevents bacteria from attaching. On the other hand, an extremely hydrophilic surface can cause water to spread out, reducing the contact area between the bacteria and the surface, which also reduces bacterial adhesion [43,44,45,46]. An extremely hydrophilic surface would have a contact angle close to 0°, while an extremely hydrophobic surface would have a contact angle close to 180° [47,48]. The SiCN film in this study does not belong to an extremely hydrophobic or hydrophilic surface, so it may not benefit in reducing bacteria attachment.

**Figure 8:**
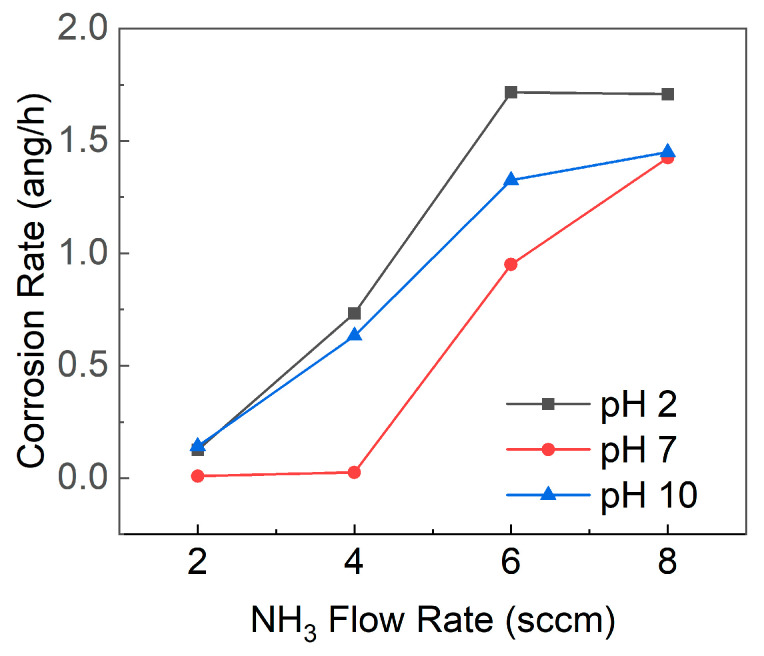
Corrosion rates of SiCN films deposited with varying NH_3_ flow rates in solutions with pH values of 2, 7, and 10.

The corrosion rate of SiCN, as depicted in Figure 8, was found to be influenced by the pH of the surrounding environment. For all SiCN films deposited at varying NH_3_ flow rates, the corrosion rate was highest at pH 2, followed by pH 10, while the corrosion rate at pH 7 was lower than at either pH 2 or pH 10. Furthermore, it was observed that a higher NH_3_ flow rate during SiCN film deposition led to increased nitrogen content in the film, resulting in a greater corrosion rate. A higher proportion of Si-N bonds relative to Si-C bonds, which may be more susceptible to hydrolysis and attack by H^+^ or OH^−^ ions, could explain this increased corrosion rate.

In highly acidic conditions (pH 2), the elevated concentration of H+ ions could accelerate the corrosion rate, as these ions react with SiCN, breaking Si-C and Si-N bonds and forming soluble silicic acid (H_4_SiO_4_) or other hydrolyzed species. In highly alkaline conditions (pH 10), the high concentration of OH- ions also may increase the corrosion rate by reacting with SiCN, breaking Si-C and Si-N bonds, and forming soluble silicate species or other hydrolyzed products. In neutral conditions (pH 7), the corrosion rate is generally lower due to the reduced concentration of aggressive ions (e.g., H^+^ and OH^−^), allowing SiCN to form a more stable and protective oxide layer on its surface [49,50]. This finding is consistent with previous studies on silicon carbide (SiC) coatings on glass-ceramic disks, where the corrosion rate followed the order pH 2 > pH 10 > pH 7 [51]. The comparable corrosion rates at pH 7 and pH 10 for an NH_3_ flow rate of 8 sccm might be attributed to the increased nitrogen content in the SiCN film due to the high NH_3_ flow rate. An elevated number of Si-N bonds, which are potentially more vulnerable to hydrolysis and ion attack, could escalate the corrosion rate. Consequently, the typically protective effect of the neutral pH 7 conditions may be undermined.

Considering that the healthy pH of saliva is approximately 7, and that acidic or basic substances such as citric or acidic solutions (e.g., Coca-Cola pH 2.45, orange juice pH 3.74, wines pH 3.34–3.68) and basic foods (e.g., spinach, soybeans, and antacids) [52,53,54,55] can alter the pH of the oral environment, the corrosion rate of SiCN films in vivo could be affected. Therefore, the corrosion rate of SiCN films under different pH conditions should be considered when evaluating their potential application as dental implant coatings.

## 4. Conclusions

This research represents a pioneering investigation into the potential application of SiCN films in dental implants, utilizing the PECVD technique for film deposition. While earlier studies have discussed the merits of SiCN for joint implants, this study breaks new ground by focusing on dental applications.

The extensive examination conducted here assessed the impact of varying NH_3_, CH4, and SiH_4_ flow rates on several critical characteristics of the SiCN films, including their chemical composition, antibacterial properties, refractive index, wettability, and corrosion rate. It was discerned that the SiCN film deposition rate increased with higher NH_3_ flow rates, producing nanometer-scale films that offer precise control over thickness, a crucial attribute for thin-film applications.

The formation of N-Si bonds, driven by the NH_3_ flow rate, emerged as a significant factor, potentially boosting the antibacterial properties of SiCN films by inducing a negatively charged surface capable of interacting with bacterial cell membranes. SiCN films with higher N content presented lower refractive indices. However, the optimal refractive index may depend on the specific dental application, necessitating a delicate balance between optical and mechanical properties.

Although the contact angle exhibited relatively minor changes, it was noted that this might not be instrumental in reducing bacterial attachment to SiCN films. The study also uncovered a correlation between the corrosion rate and pH, indicating that the acidic or basic substances in the oral environment could potentially influence the in vivo corrosion rate of SiCN films.

In conclusion, this study significantly contributes to the existing knowledge about SiCN films, particularly regarding their potential as dental implant coatings. By leveraging the PECVD technique, a promising pathway has been opened towards the development of superior dental coatings, setting the stage for further advances in dental implant technology. This research provides a strong foundation for future explorations in this domain and paves the way for the realization of the full potential of SiCN films in dental applications.

## Figures and Tables

**Table 1:** Experimental parameters and results for SiCN deposited by various flow rates.

SiH_4_ (sccm)	NH_3_ (sccm)	CH_4_ (sccm)	Si: (%)	N: (%)	C: (%)	O: (%)	Deposition Rate (nm/min)	Contact Angle (°)	Corrosion Rate (ang/hour)			Refractive Index (n)
									pH 2	pH 7	pH 10	
300	2	100	60.09	7.37	28.54	4	5.7	63.7	0.12	0.01	0.14	2.5
300	4	100	58.73	10.41	28.85	2.01	6	65.4	0.73	0.02	0.63	2.44
300	6	100	57.1	12.04	26.16	4.7	6.1	68.7	1.71	0.95	1.32	2.34
300	8	100	52.43	17.50	25.26	4.8	7	71	1.71	1.42	1.45	2.3
250	8	100	51.73	17.90	26.17	4.2	-	-	-	-	-	-
250	8	150	46.55	17.52	31.63	4.3	-	-	-	-	-	-
250	8	200	48.96	13.91	32.53	4.6	-	-	-	-	-	-
300	8	100	52.43	17.50	25.26	4.8	-	-	-	-	-	-
250	8	100	51.73	17.90	26.17	4.2	-	-	-	-	-	-
200	8	100	49.71	19.53	27.58	3.18	-	-	-	-	-	-

## Data Availability

All the data are available within the manuscript.
